# A methodology for detecting the orthology signal in a PPI network at a functional complex level

**DOI:** 10.1186/1471-2105-13-S10-S18

**Published:** 2012-06-25

**Authors:** Pavol Jancura, Eleftheria Mavridou, Enrique Carrillo-de Santa Pau, Elena Marchiori

**Affiliations:** 1 Institute for Computing and Information Sciences, Radboud University Nijmegen, Nijmegen, 6500 GL, The Netherlands; 2 Department of Medical Microbiology, Radboud University Medical Center, Nijmegen, 6500 HB, The Netherlands; 3 Department of Molecular Biology, Nijmegen Centre for Molecular Life Sciences, Radboud University Medical Center, Nijmegen, 6500 HB, The Netherlands

## Abstract

**Background:**

Stable evolutionary signal has been observed in a yeast protein-protein interaction (PPI) network. These finding suggests more connected regions of a PPI network to be potential mediators of evolutionary information. Because more connected regions of PPI networks contain functional complexes, we are motivated to exploit the orthology relation for identifying complexes that can be clearly attributed to such evolutionary signal.

**Results:**

We proposed a computational methodology for detecting the orthology signal present in a PPI network at a functional complex level. Specifically, we examined highly functionally coherent putative protein complexes as detected by a clustering technique in the complete yeast PPI network, in the yeast sub-network which spans only ortholog proteins as determined by a given second organism, and in yeast sub-networks induced by a set of proteins randomly selected. We proposed a filtering technique for extracting orthology-driven clusters with unique functionalities, that is, neither enriched by clusters identified using the complete yeast PPI network nor identified using random sampling. Moreover, we extracted functional categories that can be clearly attributed to the presence of evolutionary signal as described by these clusters.

**Conclusions:**

Application of the proposed methodology to the yeast PPI network indicated that evolutionary information at a functional complex level can be retrieved from the structure of the network. In particular, we detected protein complexes whose functionality could be uniquely attributed to the evolutionary signal. Moreover, we identified functions that are over-represented in these complexes due the evolutionary signal.

## Background

Analysing and mining protein-protein interaction (PPI) networks data using evolutionary information is a central research area in bioinformatics (see e.g. [1-10]). In this context, evolutionary information is usually described by means of the orthology relation between proteins. In general, two proteins are orthologous if they originated from a common ancestor, having been separated in evolutionary time only by a speciation event. Orthologous proteins have high amino acid sequence similarity and usually retain the same or very similar function, which allows one to infer biological information between the proteins. Obviously, orthology as such is very important in studying evolution. Therefore, the problem of establishing proper orthology relations has been widely studied in comparative genomics (see for instance [11]) and many databases and public resources of orthologs have been made available, such as Inparanoid [12,13] and OrthoMCL-DB [14].

A recent study performed by Wutchy et al [3] used such available orthology information for detecting stable evolutionary signal in a yeast PPI network. This signal was extracted at a *protein-protein interaction level*, using pairwise orthologs with respect to various different species. The authors observed that a high local clustering around protein-protein interactions correlates with evolutionary conservation of the participating proteins. This means that highly connected proteins and protein pairs embedded in a well clustered neighbourhood tend to be evolutionary conserved and therefore retain their evolutionary signal. These findings suggest also that more connected regions of a PPI network are potential mediators of evolutionary information.

Motivated by the above observations, in this paper we focus on the explicit use of orthology for detecting evolutionary signal at a *functional complex level*, that is, functional complexes that can be clearly attributed to this evolutionary signal. To this aim, we try to characterize functions of those complexes predicted by clustering the sub-graph of a PPI network induced by all proteins having orthologs in another given species, but not predicted (or predicted for a smaller fraction of proteins) neither when clustering the entire network, nor when clustering sub-graphs of the network induced by random sampling of proteins. We consider the resulting functions as a strong characterization of the underlying evolutionary signal of orthologs at functional complex level, since they are suppressed or not observed when clustering using the entire network and are not outcomes of a stochastic process.

Specifically, given the yeast PPI network and proteins from another species, we apply a state-of-the-art clustering algorithm to (1) the yeast PPI network, (2) the sub-network of the yeast PPI network induced by selecting only proteins with ortholog in the considered other organism, and (3) the sub-networks of the yeast PPI network induced by sampling a given number of proteins at random. In this way we generate three classes of clusters called *GC *(global clusters), *OC *(ortholog clusters) and *RC *(random clusters). Note that the latter class of clusters is the collection of cluster sets produced by the application of clustering to the PPI network induced by a random selection of a set of proteins (of size equal to that of the set of proteins used to generate the *OC *class) repeated for dozens times. For all clusters in each class we infer putative functions by measuring their gene ontology (GO) functional enrichment [15] using only experimentally validated annotations, and consider as putative protein complexes only those clusters with a putative function that is significantly coherent within the corresponding cluster.

The putative complexes of the *GC *class represent results globally observable in the whole interaction data without any additional information and hence play also a suppressor of any potential external biological signal present in the data. The putative complexes of the *RC *class simulate a random signal of the given protein sample size in the protein interaction data. Thus, the *OC *class complexes may be attributed to the orthology signal only when their functionality clearly differentiates from those of *GC *and *RC *class.

To this end, for a set of complexes and a certain function, we compute the functional retrieval index as the fraction of proteins contained in the complexes and having the function experimentally validated with respect to the set of candidate proteins having the function also experimentally validated and from which complexes were derived. This fraction quantifies the presence of that function in a given protein complex set. This allows us to identify functions whose proteins' fraction is higher in complexes from the *OC *class than in complexes from the other two classes. Consequently, we consider the corresponding complexes in *OC *class as describing the orthology signal (with respect to the considered species). Furthermore, we analyse those complexes in the *OC *class having a predicted function for its proteins that is not inferred when using complexes of *GC *class. Finally we discuss the new meaningful functions for well-defined as well as for unknown proteins that are present in the compilation of putative complexes.

In previous works on phylogenetic analysis of protein networks and complexes evolutionary information was usually used as a mean for evaluating the preservation of orthology information in functional modules [2,5-7]. Here, however, we incorporate evolutionary information beforehand and perform a comparative differential analysis for detecting evolutionary signal at complex, functional level. Our identification of protein complexes uses only the topology of the network of the considered species and orthology information from another species, without requiring knowledge on the interactome of the other species.

In general, our approach differs from comparative network methods [10], as the latter aim to find evolutionary conserved modules across species, thus exploiting both orthology and network topology of the considered organisms. The clusters we obtain are in one species and are related to the orthology signal with respect to another species, but are not required to be evolutionary conserved through species (we do not enforce any type of similarity at the graph-structure level). Furthermore, comparative methods mostly do not use 'known' orthologs in available databases but rather they rely on sequence similar proteins, where the level of required similarity is determined by a minimal similarity score threshold. Instead, our method exploits the orthology information available in existing databases. Moreover, the study we propose differs from those aiming to find evolutionary conserved modules across species because their output results in cluster multiplets derived from all PPI networks of multiple species being compared, where clusters contained in one multiplet are topologically and genomic similar to each other. In particular, in [16] computational technique for dividing PPI networks was proposed in order to perform modular network alignment [17]. Results indicated that aligning pairs of sub-networks from different species, where these sub-networks are obtained by expanding articulation hubs, may lead to the discovery of conserved protein modules that are not detected when aligning the two whole networks. This is different from the research question tackled in this paper, namely to detect the orthology signal at a functional complex level in a single PPI network given another species. The methodology proposed in this paper generates orthology-driven clusters that contain evolutionary functional signal but are not in general conserved across species. Indeed, this could correspond to scenarios where a functional module retains its evolutionary origin while it changed its conformation in other species after speciation, due to evolutionary (pressure) events, resulting in a cluster with some links preserved, other being created and some links being lost.

## Results and discussion

We performed the proposed computational analysis on a widely used and well-studied species, namely *Saccharomyces cerevisiae *(yeast), since its PPI network is one of the best characterized and the functionality of its proteins has been extensively studied. This makes yeast a good standard model species for protein network analysis.

Protein orthology relationships between the following pairs of organisms were considered:

• *Saccharomyces cerevisiae *vs. *Escherichia coli*

• *Saccharomyces cerevisiae *vs. *Caenorhabditis elegans*

• *Saccharomyces cerevisiae *vs. *Drosophila melanogaster*

• *Saccharomyces cerevisiae *vs. *Homo sapiens*

*Escherichia coli *(E.coli), *Caenorhabditis elegans *(worm), *Drosophila melanogaster *(fly) and *Homo sapiens *(human) are standard organisms used in protein network and genome comparative studies (e.g [18,19]) and represent the diverse life-forms from a prokaryote (E.coli) to the highly complex eukaryote (human). Yeast proteins having an ortholog in another species are here called yeast orthologs. Hence, each species comparison produces a different set of yeast orthologs to be investigated.

### Generating the cluster classes

A state-of-the-art method for detecting communities in biological networks known as MCL [20] was used for clustering networks. MCL was applied to generate the following classes of clusters:

• OYC-E - yeast clusters found using the sub-network induced by the yeast-E.coli ortholog set.

• OYC-W - yeast clusters found using the sub-network induced by the yeast-worm ortholog set.

• OYC-F - yeast clusters found using the sub-network induced by the yeast-fly ortholog set.

• OYC-H - yeast clusters found using the sub-network induced by the yeast-human ortholog set.

These groups are of the *OC *class mentioned above and we generally refer to them by the common name OYC (ortholog yeast clusters). The following classes of clusters were generated using random sampling:

• RYC-E - yeast clusters found using the sub-network induced by random sampled proteins of the same number as the number of proteins in the yeast-E.coli ortholog set.

• RYC-W - yeast clusters found using the sub-network induced by random sampled proteins of the same number as the number of proteins in the yeast-worm ortholog set.

• RYC-F - yeast clusters found using the sub-network induced by random sampled proteins of the same number as the number of proteins in the yeast-fly ortholog set.

• RYC-H - yeast clusters found using the sub-network induced by random sampled proteins of the same number as the number of proteins in the yeast-human ortholog set.

These groups belong to the *RC *class and we generally refer to them by the common name RYC (random yeast clusters). For each of the four cases given above we performed 1000 runs. Recall that every run produces one particular RYC group. In order to compare these clusters with the GYC or OYC one, we consider the average values of RYC groups computed over all 1000 simulations according to a given ortholog set.

Finally, when MCL was applied to the whole yeast network, we get clusters of the above-mentioned *GC *class, and we refer to them by the name GYC (global yeast clusters).

Table 1 reports the number of GYC, OYC and RYC clusters identified by MCL, the number of functional complexes extracted from these clusters, the average size of the clusters and of the complexes, and the percentage of the clusters that correspond to functional complexes. The number of clusters, complexes and their average size are similar for RYC and OYC, while, as expected, more clusters (hence complexes) are generated by using GYC, and their average size is slightly bigger than that of those detected using RYC and OYC. It is interesting to note that the sensitivity of the method does not change significantly with respect to the (sub-)network it is applied to, resulting in about 40% of the detected clusters being functional complexes for GYC, and in the range 37-44% for RYC and OYC. This indicates the robustness of MCL with respect to the considered sampling strategies.

**Table 1:** The number and average size of detected protein clusters and putative protein complexes.

**Clust. Gr**.	#Clusters	$\bar{\left|C\right|}$	#Complexes	$\bar{\left|C\right|}$	Ratio (%)
GYC	365	8	147	11.1	40.3%

OYC-E RYC-E	37 34.31 (±3.82)	5 4.17 (±0.40)	14 12.69 (±2.96)	5.5 5.06 (±0.68)	37.8% 37.0%

OYC-W RYC-W	181 175.22 (±7.21)	7 6.08 (±0.28)	80 67.85 (±5.87)	8.7 8.07 (±0.52)	44.2% 38.7%

OYC-F RYC-F	191 181.97 (±7.51)	7 6.15 (±0.36)	80 70.32 (±6.01)	9.33 8.19 (±0.53)	41.9% 38.6%

OYC-H RYC-H	203 196.38 (±7.80)	7 6.29 (±0.45)	90 75.71 (±6.21)	9.33 8.41 (±0.54)	44.3% 38.6%

Clust. Gr. - the cluster group (class), #Clusters - the number of clusters, #Complexes - the number of complexes, $\bar{\left|C\right|}$ - the average cluster or complex size, Ratio (%) - the percentage of clusters that are functional complexes

### Identifying orthology signal at protein complex level

The detected putative complexes are used to identify orthology-related functions. For each class of putative complexes we compute the functional retrieval indexes with respect to the protein sample set from which the complexes were derived by applying the formula (1) (see Methods). Then, for each function *f *associated with complexes of the OYC class, we compare its retrieval index *ϱ*(*f,O*) with the retrieval indexes *ϱ*(*f,R*_95%_) and *ϱ*(*f,V*) for both RYC and GYC classes using the rule (2) (see Methods).

One may consider the comparison of *ϱ*(*f,O*) with *ϱ*(*f,R*_95%_) as the *random sample filter *and *ϱ*(*f,O*) with *ϱ*(*f,V*) as the *global sample filter*. Only if *ϱ*(*f,O*) is greater than both *ϱ*(*f,R*_95%_) and *ϱ*(*f,V*), then the function *f *and with it associated OYC complexes are considered to be *orthology-related*. Application of the random sample filter differentiates the functions of the OYC class complexes from those which are likely to be observed within the complexes of class RYC and thus by chance. In the case of global sample filter it extracts functions which have greater retrieval rates within the complexes of class OYC than within the complexes of class GYC. Hence, these functions are suppressed when considering complexes present in the global topology of the PPI network and are unveiled when considering only the complexes formed by orthologs.

Table 2 reports the effect of these filters on the number of functions and associated complexes of the OYC class, when they are applied separately and when they are combined. We may observe that the global sample filter has no reduction effect on the number of complexes although from about one third to one quarter of all functions are omitted. This substantiates that indeed the complexes consisting of orthologs are well-differentiated from the complexes observed in the global topology of the PPI network.

**Table 2:** The effect of filtering procedures

**Clust. Gr**.		Total	*ϱ*(*f,R*_95%_)	*ϱ*(*f,V*)	max{*ϱ*(*f,V*),*ϱ*(*f,R*_95%_)}	Ratio (%)
OYC-E	#Complexes #Functions	14 251	14 150	14 184	14 144	100.00% 57.37%

OYC-W	#Complexes #Functions	80 767	65 124	80 526	65 123	81.25% 16.04%

OYC-F	#Complexes #Functions	80 775	68 109	80 487	68 109	85.00% 14.06%

OYC-H	#Complexes #Functions	90 735	89 79	90 444	89 78	98.89% 10.61%

Clust. Gr. - the cluster group (class), #Complexes - the number of complexes, #Functions - the number of functions, Total - the numbers in total, *ϱ*(*f,R*_95%_) - the numbers after applying the random sample filter, *ϱ*(*f,V*) - the numbers after applying the global sample filter, max{*ϱ*(*f,V*), *ϱ*(*f,R*_95%_)} - the numbers after applying the both filters, Ratio (%) - the percentage of complexes or functions which passes through the both filters

In the case of the random sample filter the number of functions drops considerably, whereas the number of complexes still remains high. As a result, when both filters are combined, one may interpret the total reduction on the number of complexes and functions as primarily caused by the random sample filter, while there is almost no effect on the reduction due to the global sample filer, especially on the number of complexes.

This suggests that in the set of all annotations associated with a given complex of the OYC class, it is very likely to observe an orthology-related function despite the sparse distribution of orthology-related functions in the GO hierarchy. As a result, more than 80% of the OYC complexes are always indeed orthology-related complexes, which suggests they mostly do not correspond to an outcome of a stochastic event.

We discuss in the sequel some interesting orthology-related functions as well as novel protein function predictions derived using the proposed methodology.

### On orthology-related functions

In the set of yeast orthologs with respect to E.coli we identified 144 orthology-related functions. Table 3 reports only higher level functions in GO hierarchy as determined by the GO slim functional terms (GO slim version: 1.1.1543, date: 19/10/2010, [21]). Each GO slim characterizes a certain type of biological functions which have some features and tasks in common, and hence they define the functional categories in a biological system.

**Table 3:** Orthology-related functional categories for yeast-E.coli orthologs

**Clust. Gr**.	GO ID	Name	GO Domain
OYC-E	GO:0005840	ribosome	CC
	GO:0005694	chromosome	CC
	GO:0000228	nuclear chromosome	CC
	
	GO:0003677	DNA binding	MF
	GO:0005215	transporter activity	MF
	
	GO:0007049	cell cycle	BP
	GO:0006811	ion transport	BP
	GO:0006519	cellular amino acid metabolic process	BP

Clust. Gr. - the cluster group (class), CC - cellular component, MF - molecular function, BP - biological process

Considering cellular compartments of a cell, we identified ribosomal and chromosomal proteins as being orthology-related. Indeed, it has been shown that the ribosomes in the mitochondria of eukaryotic cells resemble those in bacteria, reflecting the likely evolutionary origin of this organelle [22]. Considering other reported functional categories, numerous phylogenetic data provide strong evidences that there is a constant evolutionary pressure in conserving critical functional domains on proteins that are significant for cell survival. These proteins are usually components of DNA/RNA replication, transcription and translation apparatus or they are involved in ion transport processes.

Because worm, fly and human all belong to eukaryotes, we looked at their common orthology-related functions (reported in Table 4). Considering molecular functions, we retained mostly kinases activity proteins and DNA binding proteins. This is true in particular for proteins of kinase activity, which have been found conserved among eukaryotes: these kinase' functional conservations were investigated for yeast, worm, fly and human when studying their evolution [23]. Orthology-related DNA binding proteins have been also known to exhibit high sequence conservation among eukaryotes (e.g [24,25]).

**Table 4:** Orthology-related functions for yeast-worm, yeast-fly, and yeast-human orthologs

**Clust. Gr**.	GO ID	Name	GO Domain
	GO:0042555	MCM complex	CC
	
	GO:0004672	protein kinase activity	MF
	GO:0004674	protein serine/threonine kinase activity	MF
	GO:0003883	CTP synthase activity	MF
	GO:0043565	sequence-specific DNA binding	MF
	
	GO:0009987	cellular process	BP
	GO:0044257	cellular protein catabolic process	BP
	GO:0051603	proteolysis involved in cellular prot. catab. proc.	BP
OYC-W	GO:0019941	modification-dependent protein catabolic process	BP
OYC-F	GO:0006511	ubiquitin-dependent prot. catab. proc.	BP
OYC-H	GO:0006220	pyrimidine nucleotide metabolic process	BP
	GO:0009147	pyrimidine nucleoside triphosph. metab. proc.	BP
	GO:0006221	pyrimidine nucleotide biosynthetic proc.	BP
	GO:0009218	pyrimidine ribonucleotide metabolic proc.	BP
	GO:0009208	pyrimidine ribonucleoside triphosph. metab. proc.	BP
	GO:0009148	pyrimidine nucleoside triphosphate biosynth. proc.	BP
	GO:0009220	pyrimidine ribonucleotide biosynth. proc.	BP
	GO:0009209	pyrimidine ribonucleoside triphosph. biosyn. proc.	BP
	GO:0046036	CTP metabolic process	BP
	GO:0006241	CTP biosynthetic process	BP

Clust. Gr. - the cluster group (class), CC - cellular component, MF - molecular function, BP - biological process

Regarding the Mcm complex, it consists of six eukaryotic Mcm proteins which also share significant sequence similarity with one another. These proteins serve as the eukaryotic replicative helicase, the molecular motor that both unwinds duplex DNA and powers fork progression during DNA replication [26] and therefore are expected to be orthology-related.

CTP and pyrimidine processes are incorporated in the growth of RNA and DNA during the process of transcription or DNA replication. Short-term energy storage is also one of the functions of pyrimidines. Hence, as mentioned above, there is a pressure on evolutionary conservation of these processes vital for a cell survival. Last but not least, proteins involved in ubiqiunting-dependent processes contain a highly conserved ubiquitin-conjugating (UBC) domain; thus, the function is also orthology-related.

### On orthology-related complexes and novel predictions

Orthology-related complexes are those complexes of the OYC class whose proteins perform at least one orthology-related function. In addition, we call *unique complexes *those complexes whose proteins have a predicted function that is not inferred for those proteins by any GYC complex. These are the complexes that are new and derived using (the protein complex composition present in) the orthology sub-network, that is, uniquely linked to the orthology signal.

Given a unique cluster and its protein having a new predicted function not inferred by any GYC complex containing the protein. Then, if the function prediction is experimentally or computationally annotated in SGD, this prediction is verified. Analogously, if we find the new predicted function has not been experimentally or computationally annotated in SGD, then this prediction is indeed a novel prediction. Observe that one cluster can have verified as well as novel predictions at the same time. The number of orthology-related complexes as well as those which produce verified and/or novel protein function predictions are reported in Table 5. We may observe that, for each ortholog set, from all complexes with a novel prediction, more than 80% are orthology-related complexes. This is analogous to the reduction effect on the whole set of complexes mentioned above.

**Table 5:** The numbers of putative protein complexes containing unique, verified and novel protein function predictions

**Clust. Gr**.		Total	Unique	Verified	Novel
OYC-E	#All #Ort.-related	14 14	13 13	1 1	13 13

OYC-W	#All #Ort.-related	80 65	69 57	12 11	63 52

OYC-F	#All #Ort.-related	80 68	62 52	12 11	59 50

OYC-H	#All #Ort.-related	90 89	72 72	10 10	68 68

Clust. Gr. - the cluster group (class), #All - the number of all complexes, #Ort.-related - the number of orthology-related complexes

Examples of novel orthology-related complexes are given in Table 6: they demonstrate that by examining different sets of orthologs we found specific putative complexes, most of them crucial for a living cell.

**Table 6:** Novel orthology-related complexes

Cluster ID	Proteins	Prediction	Cluster Group
Cluster 1	ATP1		OYC-E
	ATP2	mitochondrial proton-transporting ATP synthase, catalytic core	OYC-E
	ATP3		OYC-E

Cluster 2	MMS2		OYC-W,OYC-F,OYC-H
	UBC13	ubiquitin conjugating enzyme complex	OYC-W,OYC-F,OYC-H
	ERR3		OYC-W,OYC-F,OYC-H

Cluster 3	UBC7		OYC-W,OYC-F,OYC-H
	UBC5		OYC-W,OYC-F,OYC-H
	UBC6		OYC-W,OYC-F,OYC-H
	UBC1	protein ubiquitination	OYC-W,OYC-F,OYC-H
	UBC8		OYC-W,OYC-F,OYC-H
	UBC4		OYC-W,OYC-F,OYC-H
	VIP1		OYC-W,OYC-F,OYC-H

Cluster 4	SEC22		OYC-W,OYC-F,OYC-H
	SFT2		OYC-W,OYC-F,OYC-H
	SED5		OYC-W,OYC-F,OYC-H
	BET3		OYC-W,OYC-F,OYC-H
	SLY1		OYC-W,OYC-F,OYC-H
	SEC17	Golgi vesicle transport, Golgi apparatus	OYC-W,OYC-F
	SEC18		OYC-W,OYC-F
	COS1		OYC-F,OYC-H
	SYM2		OYC-F,OYC-H
	GPA1		OYC-W
	STE4		OYC-W
	AKR1		OYC-W
	YKT6		OYC-W

Cluster 5	SEC23		OYC-W,OYC-F,OYC-H
	SEC24		OYC-W,OYC-F,OYC-H
	SFB2	COPII vesicle coat	OYC-W,OYC-F,OYC-H
	HIP1		OYC-W,OYC-F,OYC-H
	GRH1		OYC-W,OYC-F
	BUG1		OYC-F

Cluster 6	SEC9		OYC-W,OYC-H
	SNC1		OYC-W,OYC-H
	SNC2	SNARE complex, plasma membrane	OYC-W,OYC-H
	SSO1		OYC-W,OYC-H
	SSO2		OYC-W,OYC-H

Cluster 7	ATG5		OYC-F
	ATG7	C-terminal protein lipidation	OYC-F
	SSO2		OYC-F

Cluster 8	HXT3		OYC-E,OYC-F
	HXT2		OYC-E,OYC-F
	HXT4		OYC-E,OYC-F
	HXT1	hexose transmembrane transporter activity	OYC-E,OYC-F
	SNF3		OYC-E,OYC-F
	RGT2		OYC-E,OYC-F
	CYC8		OYC-E

For instance, proteins of Cluster 1 are predicted to be involved in mitochondrial proton transporting ATP synthase, catalytic core. While ATP1 and ATP2 are indeed the part of the catalytic core, ATP3 is part of the central stalk of mitochondrial proton-transporting ATP synthase. Cluster 1, however, gives a proper suggestion for the mechanism of the ATP3. Moreover, as ATP3 interacts with ATP2 it may be involved also in the catalytic core.

Cluster 2 and 3 are ubiquintin complexes. In general, in eukaryotes ubiquitin-dependent processes relate to protein degradation, because it is catalysed by a family of ubiquitin-carrier enzymes (E2) which contain a highly conserved ubiquitin-conjugating (UBC) domain. Previous reports showed that numerous members of this family are functionally overlapping [27,28]. Hence, as one could expect, our complexes, Cluster 2 and Cluster 3, are found for all eukaryotic yeast's orthologs, consisting of ubiquitin-conjugating enzymes that mediate protein degradation, indicating a highly conservation of UBCs during evolution for eukaryotes. In Cluster 2 ERR3 is a protein of unknown function, which has similarity to enolases. This suggests that ERR3 is part of the ubiquitin conjugating enzyme complex. In case of Cluster 3 the VIP1 was the only protein found with no UBC activity indicating the involvement of kinases in the complex process of ubiquitination. However, experimental data demonstrated the ubiquitin-proteasome machinery to control the levels of kinases by proteolysis [29]. As the mechanism of ubiquitin-mediated protein degradation is poorly understood it requires further investigation.

Next, we discuss proteins in the closely related complexes Cluster 4 and Cluster 5. The protein families that mediate vesicle trafficking are conserved through phylogeny from yeast to human, as well as throughout the cell from the endoplasmic reticulum to the plasma membrane [30]. Our analysis showed proteins of the SEC family (SEC22, SEC23 and SEC24) and others as SED5, BET3, SLY1, HIP1 and SFB2 conserved from worm to human and involved in the coat protein complex II (COPII) that selectively transport molecules and vesicle fusion proteins from the endoplasmic reticulum (ER) to the Golgi complex [31]. Other proteins included in the complexes but not for all species as BUG1 and GRH1 have been observed co-localizing on the cis-Golgi and they form a heterooligomeric complex binding GRH1 at the well conserved C terminus of BUG1 [32]. The role of these two proteins in ER to Golgi transport is mediated by the interaction between GRH1 with the SEC23/24 complex, proteins that we could identify in the same complex a that of GRH1 and BUG1. In these clusters related with vesicle trafficking we could observe other proteins like SFT2, COY1 and GOS1 not annotated for the ER to Golgi vesicle-mediated transport term but we could classify them in the correct cluster. These proteins have been observed to be required for vesicle fusion with the Golgi complex [33,34].

Further interesting outcomes are Cluster 6 and Cluster 7. Both clusters share the SSO2 protein but they produce different functional predictions. In the case of Cluster 6 SSO2 interacts with proteins of the yeast SNARE complex (SEC9, SNC1, SNC2, SSO1), the core of the machinery required for membrane fusion, while in Cluster 7 SSO2 is involved with the Cvt pathway proteins (ATG5, ATG7), a biosynthetic transport route for a distinct subset of resident yeast vacuolar hydrolases. Reggiori et al [35] described that the biogenesis of Cvt vesicles apparently requires a fusion step catalysed by the VFT tethering factor and by the SNARE complex but they failed to show the proteins that are related in the interaction between the Cvt pathway and the SNARE complex. Although to further elucidate the real role of the SSO2 protein in the interaction between the Cvt pathway and the SNARE complex an experimental validation is necessary, these results show the capability of the presented methodology not only to classify proteins interacting within the same or related clusters but also to predict unknown protein interactions between different pathways and complexes that are currently under investigation. Interestingly, Cluster 6 is found in OYC-W and OYC-H cluster groups corresponding to yeast-worm and yeast-human ortholog sets while Cluster 7 is found only in OYC-F corresponding to the yeast-fly ortholog set suggesting the complexity and versatility of protein complex evolution.

Finally, we discuss Cluster 8, which contains the novel prediction for the SNF3 and RGT2 proteins. This complex was observe for yeast-fly and yeast-E.coli ortholog sets but not for the other ones. Previous studies in yeast demonstrated that SNF3 and RGT2 are integral membrane proteins with unusually long carboxy-terminal tails involved in glucose transport. This is in compliance with our results that showed both proteins to have a glucose transport activity. However, according to recent studies, although both proteins are very similar to glucose transporters, they apparently do not transport glucose but they interact as glucose sensors. Özgan et al [36] demonstrated that glucose signalling is not the result of glucose transport and that the C- termini of both proteins are signalling domains of these glucose sensors. Nonetheless, it remains unclear how glucose transport is regulated and therefore our prediction can be considered as valid. In addition, as the SNF3/FGT2 protein interaction was not found in yeast ortholog set with respect to human and worm, it indicates that the protein complex is not conserved among all species. Aside the SNF3/RGT2 complex the predicted cluster includes also the HXT-transporters which are responsible for glucose uptake. Moreover, in OYC-E this protein complex was assembled with the contribution of CYC8, a yeast protein that binds to the promotors of the HXT genes blocking their transcription. This finding is very interesting as E.coli contains no nucleus and therefore it is likely that an equivalent protein complex exists.

## Conclusions

We proposed a novel methodology for quantifying the functionality of the orthology signal in a PPI network at a functional complex level. The methodology performs a differential analysis between the functions of those complexes detected by clustering a PPI network using only proteins with orthologs in another given species, and the functions of complexes detected using the entire network or sub-networks generated by random sampling of proteins.

Results of our experimental analysis indicated the usefulness of the proposed methodology to identify functional categories and complexes that can be clearly attributed to the presence of an evolutionary (orthology) signal, as supported by biological evidence from related studies.

As a future work, we intend to investigate possible extension of the methodology to increase its sensitivity. In particular one can exploit the inheritance property present within the GO hierarchy, namely each filial GO term may inherit features of its parental terms. For example, one could propagate the evolutionary signal between the two closest orthology-related function in the GO hierarchy such that all GO terms present on the paths between these two terms are also orthology-related.

## Methods

### Data

The analysis was performed on the budding yeast interaction data collected by Georgii et al [37]. The data combines interaction data from DIP [38] and MPact [39], and interactions from the core datasets of the TAP mass spectrometry experiments [40,41]. This yeast interaction data are weighted by the method proposed by Jansen et al [42] to measure the confidence of interactome. As a result, the low confidence interactions are ignored and the final yeast PPI network consists of 3545 proteins and 14354 interactions.

For obtaining orthology information we used the Inparanoid Database of Pairwise Ortholog [13,43]. This database contains clusters of ortholog groups (COGs) constructed by the Inparanoid program [44], which is a fully automatic method for finding orthologs and in-paralogs between two species. Ortholog clusters in the Inparanoid are seeded with a two-way best pairwise match (the seed ortholog pair), after which an algorithm for adding in-paralogs is applied. Because in-paralogs are homologs that arise when duplication occurs after speciation, and the duplicated gene often still retains the function of the ortholog [45], they should be likely found in one protein complex. Therefore we consider all proteins present in COGs for inducing an orthology PPI sub-network and, for simplicity, we consider all proteins in a COG as orthologs. Specifically, in this study we call orthologous protein or ortholog a protein which is a part of an orthologous cluster produced by the Inparanoid when comparing two species.

In our analysis, COGs were obtained for the following pairs of organisms:

• *Saccharomyces cerevisiae *versus *Escherichia coli*

• *Saccharomyces cerevisiae *versus *Caenorhabditis elegans*

• *Saccharomyces cerevisiae *versus *Drosophila melanogaster*

• *Saccharomyces cerevisiae *versus *Homo sapiens*

Yeast proteins in the derived ortholog groups are called yeast orthologs. We considered the following 4 sets of yeast orthologs (present in the yeast PPI data), namely *yeast-E.coli, yeast-worm, yeast-fly, yeast-human*, consisting of 451, 1664, 1724, and 1850 proteins, respectively.

### Quantifying orthology signal

We are interested in quantifying the orthology signal by means of a set of functions of those putative protein complexes detected by applying a clustering algorithm to a PPI network. To this end, we directly exploit evolutionary information of proteins as described by the presence of orthologs in another, given species. We call these proteins 'true orthologs'. The following terminology is used in the sequel. A PPI network is represented by means of a graph *G*(*V*, *E*), where *V *is the set of nodes (proteins) and *E *is the set of edges (binary interactions). Let *X *be a subset of nodes *V *(e.g. ortholog set). The set *X *induces a sub-graph *G*[*X*] = (*X*, *E_X_*) of *G*, with set *X *of nodes and set *E_X _*of those edges of *E *that join two nodes in *X*. For a set *S*, we denote by |*S*| the number of its elements.

Given a PPI network *G *= (*V*, *E*) and a given species *s*, we propose a methodology for detecting the orthology signal at a functional complex level, consisting of the following steps.

1*. Retrieve from a database the set O of 'true orthologs' of V with respect to s, with *|*O*| = *n*.

2. *Generate the following three classes of clusters, using a given clustering algorithm*.

*(a) Class 1 clusters (GC). Apply clustering to the whole PPI network G*.

*(b) Class 2 clusters (OC). Apply clustering to the sub-network induced by O*.

*(c) Class 3 clusters (RC). Apply clustering to the sub-network induced by a randomly selected subset of V of size n. Repeat the process a number N of times. Consider all sets of clusters detected across these runs (RC *= {RC_1_, RC_2_,...,RC_N_}).

3. *For each class of clusters*,

*(a) Infer putative complexes and identify their functions*.

*(b) For each identified function, compute its retrieval index as the fraction of those proteins in the detected complexes which have been assigned to that function and experimentally verified to have that function*.

4. *Select the set of those functions derived using putative complexes from class OC and whose fractions are higher than those of the same function derived using putative complexes from class GC and from class RC*.

5. *Output the set of putative complexes from class OC having at least one of the selected functions*.

The set of putative complexes of class *GC *represent results of no selection (global or suppressor) bias and the collection of the sets of putative complexes present in the class *RC *corresponds to the random selection bias. Accordingly, the complexes of class *OC *represent the orthology selection bias. Thus, the method considers the complexes exhibiting orthology signal as those of the *OC *class having a function which may not be attributed neither to the global bias nor to the random bias.

Next, we discuss the details of main steps of the proposed methodology.

#### Generating the cluster classes

Each class of clusters is produced by applying a clustering technique to the corresponding PPI (sub-)network. In this study we used the MCL clustering. MCL [20] computes clusters based on simulation of stochastic flow in graphs and it is widely used on many domains. It is able to use information on weights of edges of a given network if available. A first successful application of this algorithm on biological networks was presented in [46]; MCL was also modified for detecting orthologous groups [47]. A recently published comparative study [48] indicated that MCL outperforms other algorithms for clustering PPI networks. The inflation parameter of the algorithm was set to 1.8 as suggested in [48].

#### Inferring putative complexes and their functionalities

We want consider putative protein complexes containing more than a single protein-protein interaction. Therefore, after applying the clustering method we retain only clusters of size greater than or equal to 3. In order to infer the putative functions of a cluster, we measured the enrichment of functional annotations of the corresponding protein set, as entailed by the GO annotation [15], using one of the well-established tools, the Ontologizer [49,50]. The Ontologizer offers various algorithms for measuring GO enrichments. Here, we apply the standard statistical analysis method based on the one-sided Fisher's exact test [49], which measures the statistical significance of an enrichment and assigns to the cluster a p-value for each enriched function. The p-value is further corrected for multiple testing by means of a Bonferroni correction procedure.

The GO is known to have a hierarchical structure (directed acyclic graph) which can be used to define the level of an annotation. Specifically, the level of an annotation is equal to the length of the furthest path from the root of GO hierarchy to that annotation. This strategy always defines a filial annotation to have a higher level (deeper in the hierarchy) than its all parental annotations and hence no inconsistency on the description of GO hierarchical level (a parent having the same or higher level than its child) is introduced. The GO terms closer to the root of GO give more general description of biological functions while terms closer to the leaves of GO have granular and very specific biological definitions.

To measure functional annotation enrichments of proteins present in a cluster we used only experimentally verified annotations as reported in the yeast gene association file of Saccharomyces Genome Database (SGD) (SGD version: 1.1523, date: 11/13/2010, [51]), available at the GO database (GO version: 1.1.1602, date: 16/11/2010, [21]). We excluded all computationally assigned annotations to yeast proteins to avoid introducing a possible bias, because many of these techniques use protein structure or sequence similarity which may often refer to orthology.

Each detected cluster is a potential protein complex. The quality of a protein cluster is given by the coherence of biological functions of proteins contained in the cluster. If a certain subset of proteins in a cluster has a significantly coherent function, a prediction of that function for all proteins in the cluster can be made. Note that one may obtain more than one protein function prediction if more significantly coherent functions in the cluster are found. We say that proteins of a cluster have a *significantly coherent function *or functional GO annotation if the following criteria are satisfied:

1. the GO annotation is significantly enriched by the proteins in the cluster (p-value < 0.001).

2. more than half of the proteins in the cluster has this significant annotation.

3. the annotation is at least at the GO level four from the root of GO hierarchy.

In such a case the cluster can be used as protein function predictor and the significantly enriched GO annotation of the cluster is used to predict protein function of each of the proteins in that cluster. If a cluster does not satisfy the above conditions, no prediction can be made. Similar criteria were used by, e.g. [16,52]. The condition on GO hierarchy guarantees that the prediction about biological functions is sufficiently specific and informative [53]. Each cluster which is a predictor defines a *putative protein complex *and the set of significantly coherent functions defines the set of inferred functions.

In the last step, for each putative complex we do an additional inference analogous to the protein function annotation procedure as follows. The GO hierarchy defines a parent-child relationship between GO functional terms where each descendant inherits all features of its ancestors. As a consequence, once a protein has a GO term annotation assigned, the protein has implicitly also annotations of all parental terms of the annotated function. Hence, using the same ratio, given the set of inferred functions of a putative protein complex, the complex also inherits all parental GO terms of the inferred functions. Thus, we may distinguish the following two sets of annotations, the most granular, filial, annotations, where no parent-child relationship between corresponding GO terms may be observed, and the set of all annotations which is the union of the filial annotations and all its ancestral annotations in GO hierarchy. Notice, by the definition, for a given complex all filial annotations are significantly coherent functions of the complex while the parental annotations need not to be significantly enriched.

#### Estimating the retrieval index of GO functions

Having a set or class of putative protein complexes, one can quantify, at a fine-grained, protein level, a so called retrieval index of functions inferred by the protein complexes and defined as follows.

Consider a PPI network *G*(*V*, *E*) and let *X *⊆ *V*. Let *G*[*X*] = (*X, E_X_*) be the corresponding induced sub-graph of *G *and *X*_0 _⊂ *X *be the set of singletons in *G*[*X*]; that means there is no edge (interaction) in *E_X _*containing any of the proteins in *X*_0_. We define the set of background proteins as *B*(*X*) = {*X *\ *X*_0_} and we denote *S*(*X*) the set of all proteins contained in putative complexes discovered in *G*[*X*]. Additionally, let *C*(*f*) ⊆ *V *be the set of candidate proteins for function *f*, that is, the set of all proteins having either experimentally annotated function *f *or an experimentally annotated function that is a descendant function of *f *in the GO hierarchy. Then let *P*(*f,U*) = {*U*∩*C*(*f*)} be the set of those proteins which have an experimental evidence for the function *f *and are present in the set *U *⊆ *V *. We can define *the retrieval index of a function f *in *X *as the following fraction:

(1) $$\varrho \left(f,\;X\right)=\frac{\left|P\left(f,S\left(X\right)\right)\right|}{\left|P\left(f,B\left(X\right)\right)\right|}.$$

This fraction measures the retrieval of a given function *f *from a protein sample *X *by the set of putative complexes identified in *X*. It can be viewed as an index measuring how likely a given function is present in a given set of putative complexes with respect to a given set of proteins.

Note that *ϱ*(*f,V*) corresponds to the retrieval index of *f *for the *GC *class, *ϱ*(*f,O*) corresponds to the retrieval index of *f *for the *OC *class, and *ϱ*(*f,R_i_*) corresponds to the retrieval index of *f *for the *RC_i _*∈ *RC *class, where *R_i _*is the random protein sample used at the run *i *when building the *RC *class.

#### Identifying orthology-related functions and complexes

We consider a function *f *to be related to the orthology signal if it satisfied two conditions: (a) it has a higher retrieval (at the level of putative protein complexes) in the set of orthologs than in the set of proteins of whole network and (b) it is unlikely to be retrieved when using random sampling. The second condition is formalized by comparing *ϱ*(*f,O*) with the 95th percentile of the set of retrieval indexes of *f *in the *RC *class. Specifically, for each function *f *from the GO hierarchy such that *f *∈ *B*(*O*), we compute its functional retrieval indexes for *GC*, *OC *and the *RC *classes. Then, the *function f *is *orthology-related *iff

(2) $$\varrho (f,\;O)>\max\{\varrho (f,\;V),\;\varrho (f,\;R_{95\%})\},$$

where *R*_95% _is a random protein sample *R_i _*such that ϱ(*f*, *R_i_*) is the up 95th percentile of the all *ϱ*(*f,R*_1_),...,*ϱ*(*f,R_N_*).

Finally, if a putative complex of the *OC *class has at least one orthology-related function, we consider that complex to be orthology-related.

## List of abbreviations used

PPI: protein-protein interaction; SGD: Saccharomyces Genome Database; GO: gene ontology; COGs: clusters of ortholog groups; UBC: ubiquitin-conjugating; ER: endoplasmic reticulum; COPII: coat protein complex II; *GC*: global clusters; GYC: global yeast clusters; *OC*: ortholog clusters; OYC: ortholog yeast clusters; OYC-E: ortholog yeast clusters with respect to E.coli; OYC-W: ortholog yeast clusters with respect to worm; OYC-F: ortholog yeast clusters with respect to fly; OYC-H: ortholog yeast clusters with respect to human; *RC*: random clusters; RYC: random yeast clusters; RYC-E: random yeast clusters as given by the number of yeast orthologs with respect to E.coli; RYC-W: random yeast clusters as given by the number of yeast orthologs with respect to worm; RYC-F: random yeast clusters as given by the number of yeast orthologs with respect to fly; RYC-H: random yeast clusters as given by the number of yeast orthologs with respect to human.

## Competing interests

The authors declare that they have no competing interests.

## Authors' contributions

PJ carried out the computational analysis. PJ and EM^1 ^conceived and designed the study. EM^2 ^and ECSP performed the biological interpretation of the results. All authors read and approved the final manuscript.
